# Automated Light Transmission Aggregometry with and without Platelet Poor Plasma Reference: A Method Comparison

**DOI:** 10.1055/s-0043-1762588

**Published:** 2023-02-22

**Authors:** Ulrich J. Sachs, Lida Röder, Nina Cooper, Christian Radon, Hans-Jürgen Kolde

**Affiliations:** 1 Department of Thrombosis and Haemostasis, Giessen University Hospital, Giessen, Germany; 2 Institute for Clinical Immunology, Transfusion Medicine, and Haemostaseology, Justus Liebig University, Giessen, Germany; 3 Behnk Elektronik, Norderstedt, Germany; 4 Consulting Diagnostics, Seefeld, Germany

**Keywords:** light transmission aggregometry, platelet function, precision, reproducibility, reference ranges

## Abstract

**Background** Light transmission aggregometry (LTA) is considered the gold standard for the evaluation of platelet function but is labor-intensive and involves numerous manual steps. Automation may contribute to standardization. Here, we evaluate the performance characteristics of a new automated instrument, Thrombomate XRA (TXRA), and compare it against a manual instrument (PAP-8).

**Materials and Methods** Leftover blood samples from blood donors or patients were tested in parallel with identical reagents and in identical concentrations both manually using PAP-8 and automated on the TXRA. In addition to precision and method comparison, an additional evaluation was performed on the TXRA against “virtual” platelet-poor plasma (VPPP) based on artificial intelligence. The main focus was on comparing the maximum aggregation (MA%) values.

**Results** Precision for MA% ranged from 1.4 to 4.6% on TXRA for all reagents. Normal ranges for 100 healthy blood donors on both instruments were in a similar range for all reagents, with a tendency to slightly higher values with TXRA. Most agonists resulted in normally distributed MA%. Comparing 47 patient samples on both devices showed a good correlation for both slope and MA% with some differences in individual samples with epinephrine and TRAP. Correlation between the TXRA measurement against PPP and “virtual” PPP demonstrated excellent correlation. Reaction signatures of both devices were very similar.

**Conclusion** TXRA provides reproducible LTA results that correlate with an established manual method when tested against PPP or VPPP. Its ability to perform LTA only from platelet-rich plasma without requiring autologous PPP simplifies LTA. TXRA is an important step not only for further standardizing LTA but also for a more widespread use of this important method.

## Introduction

Platelets are essential in hemostasis. 1 Because congenital and, more often, acquired platelet disorders can cause bleeding, assessing platelets is an important aspect in the laboratory work-up of patients presenting with hemorrhages. 2 Whereas full blood counts and platelet parameters such as, median platelet volume, are fully automated and therefore easy to deliver by the laboratory, platelet function studies are requested much less frequently, often only after other hemorrhagic disorders have been excluded. 3 This is most likely a consequence of the technical challenges associated with platelet function studies, but probably also the lack of standardization, and the difficulties in interpreting the results. 4 5 6 7 8 Although newer methods are in the market, many experts consider light transmission aggregometry (LTA) as the gold standard for the assessment of platelet function, introduced by Born and O' Brien in 1962. 9 10 LTA determines platelet aggregation in platelet-rich plasma (PRP) by assessing the change in light transmission in response to added specific platelet agonists. Under constant stirring, agonists, through the activation of specific receptors, can prompt platelet granule secretion, activation, and aggregation. The reactivity pattern with various agonists acting through different receptors and signaling pathways helps to establish a diagnosis. 11 However, limitations of LTA are obvious: it requires large volumes of blood for the preparation of PRP and platelet-poor plasma (PPP), which is necessary for calibrating the measurement zero. Numerous preanalytical variables including, venipuncture, centrifugation, adjustment of platelet count, and limited sample stability may affect the test outcome. 12 13 14 Instrument-related variables such as stirring intensity, physical properties of the optical system, and software settings used to calculate the various parameters of turbidity signature, as well as type, quality, and concentration of agonists further affect the test outcome. In addition, LTA remains a time-consuming test method because it includes multiple manual steps, which are also potential source of errors.

An expert panel of the Platelet Physiology Scientific and Standardization Committee (SSC) of the International Society on Thrombosis and Haemostasis (ISTH) and others have proposed steps for standardization. 14 15 16 17 18

Like in other areas of laboratory testing, automation could play a key role in improving the standardization of the procedure. A novel fully automated device for LTA, Thrombomate XRA (TXRA), was developed recently with the goal of minimizing variables. A novel feature is the ability of TXRA to measure LTA in PRP not only against autologous PPP but also against a virtual reference. In this study, the performance characteristics of the TXRA device were compared against a standard LTA instrument using fixed concentrations of agonists of the identical source on both instruments.

## Materials and Methods

### Blood Samples

The study was conducted at Giessen University Hospital, Giessen, Germany. Anonymized left-over material from healthy blood donors, patients with suspected hemostatic disorders, and patients on aspirin and/or adenosine diphosphate (ADP) receptor antagonists was used. Approval from the local ethics committee was obtained. There was no patient selection process. If LTA testing was requested from the laboratory and appropriate amounts of material were available to run both assays, subsequent samples obtained from patients taking antiplatelet drugs or from patients with a suspected bleeding disorder were included. Blood was drawn with Safety–Multifly cannulas (Sarstedt, 21Gx3/4''TW, 0.8 × 19 mm) into 10 mL Sarstedt Monovettes with 0.106 mol/L Na-citrate (9 vol blood + 1 vol anticoagulant). PRP was obtained by centrifugation for 10 minutes at 150 g, and PPP was obtained by centrifugation for 20 minutes at 1,500 g. PRP was allowed to rest for 30 minutes prior to analysis. All function studies were performed without prior adjustment of platelet counts.

### Instruments and Reagents

Test samples were run in parallel on two instruments, the automated TXRA (Behnk Elektronik, Norderstedt, Germany) and the laboratory's standard instrument, PAP-8 (möLab, Langenfeld, Germany). The same technical assistant performed all preparation and measurement steps on both instruments throughout the study.

TXRA is a stand-alone analyzer for LTA, designed for the use either with or without PPP as the reference. TXRA uses CE-marked reagent combinations provided as a unit. In this study, ADP, arachidonic acid (ARA), epinephrine (EPI), collagen (COL, fibrillary collagen from horse tendon), thrombin-receptor activating peptide (TRAP) and ristocetin (RISTO) were used. Identical reagents and reagent concentrations were used on both instruments. Concentrations were in accordance with the SSC/ISTH recommendations ( Table 1 ). 16 Low RISTO (0.6 mg/mL) was included for the characterization of suspected type 2B von Willebrand syndrome (VWS) or platelet-type VWS. TXRA automatically counts down the recommended resting time of PRP after centrifugation. 16 Subsequently, PRP is inverted in the closed sample tube for gentle standardized homogenization before it is automatically dispensed after cap-piercing into prewarmed cuvettes. The automated addition of reagents starts the test. A mechanically added steel ball mixes the sample. Using TXRA standard settings, the reaction is followed simultaneously in five measuring channels for 6 minutes by high-precision bichromatic (620 and 405 nm) light-emitting diode (LED) optics. The dual wavelength LED optical system is also used for checking samples for potential interferences induced by hyperbilirubinemia, lipemia, or hemolysis.

**Table 1:** Precision data of MA%

	TXRA		PAP-8	
Reagent	Mean CV	Range	Mean CV	Range
TRAP (10 µM)	2.1	1.1–3.0	2.1	1.1–3.0
ADP (2.5 µM)	1.6	0.9–2.0	3.7	2.6–4.2
COL (2 mg/mL)	1.4	0.7–1.9	3.0	1.0–5.0
ARA (1 mM)	4.6	2.6–6.7	3.3	1.3–6.2
EPI (5 µM)	2.2	1.3–3.9	2.8	1.9–3.7
RISTO (1.2 mg/mL)	2.1	1.2–3.1	1.7	0.6–2.8
CV (%)	2.8		3.3	
SD	1.14		4.6	

Abbreviations: ADP, adenosine diphosphate; ARA, arachidonic acid; COL, collagen; CV, coefficient of variation; EPI, ephinephrine; MA%, maximum aggregation; PPP, platelet poor plasma; RISTO, ristocetin; SD, standard deviation; TRAP, thrombin receptor activating peptide (Ser-Phe-Leu-Leu-Arg-Asn); TXRP, Thrombomate XRA.

Note: Precision was calculated using five different donors and fivefold determination for each reagent.

On TXRA, the maximum aggregation in percent for a sample is calculated according to the formula:



with *E*_base_ = basic absorbance and *E*_PPP_ = absorbance of platelet-poor plasma.

The slope is an interpolated value that describes which aggregation value would be reached if the aggregation was continued as at the point with the maximum slope.

The slope *m* of the two absorbance values *E _a_* and *E _b_* is calculated according to the formula *E _slope_* = *E* + (60 * *m* ) with



where *E* is the mean absorbance ( *E* ) and *T _a_* and *T _b_* are the time points of the two measured values, whereas *E* is the measured value in the middle between *T _a_* and *T _b_* and can be calculated by searching the value with the nearest time point to *T _a_* + ( *T _b_* − *T _a_* )/2 is found.

### Virtual Platelet-Poor Plasma Reference

The LED optical system on the TXRA was further employed for calculating the “virtual platelet-poor plasma” (VPPP) optical properties by a novel proprietary algorithm based on artificial intelligence. The VPPP value is used to “blank” the individual PRP, thereby eliminating the requirement for autologous PPP. In this study, all measurements were made against autologous PPP. The results of the VPPP method were calculated from the stored PRP reaction data in the database.

### Statistics

Precision testing was performed with five different healthy control samples, reference values were assessed using 100 different healthy control samples, and a comparison of patient data was performed using 47 different patient samples. Data were analyzed with MS Excel and Abacus statistical software (LABanalytics GmbH, Jena, Germany). Method comparisons were made with the method of Passing and Bablok or by linear regression. Correlation coefficients were calculated according to Pearson.

## Results

### Precision

**Fig. 1:**
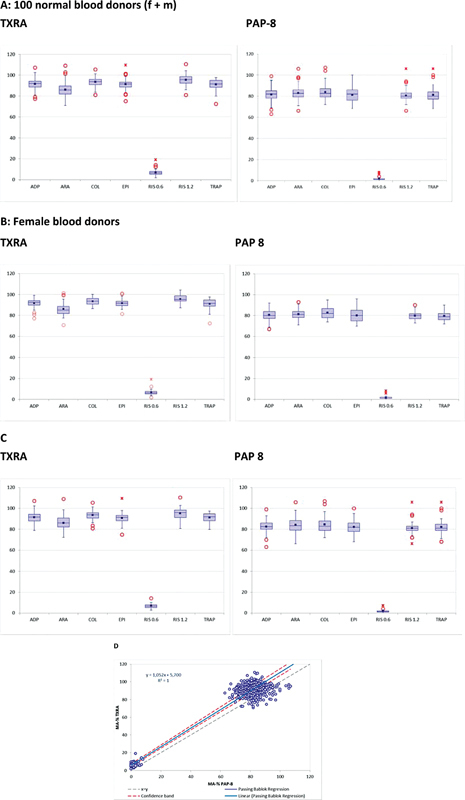
Maximum aggregation (MA%) of normal blood donors. ( **A–C** ) Distribution of MA% values obtained in ( **A** ) 100 healthy blood donors, separated in ( **B** ) *n* = 50 females and ( **C** ) *n* = 50 males on TXRA with PPP as a reference. Box-and-whisker diagrams show minimum, lower quartile, median, upper quartile, and maximum values. Outliers are indicated by circles. Crosses indicate data points outside the 2SD-range, ( **D** ) method comparison (Passing–Bablok analysis) for MA% values between TXRA and PAP-8 of 100 healthy blood donors (50 f/ 50 m).

Precision testing comprised the fivefold analysis of PRP from five different individuals with all reagents on both instruments. The results of the maximum aggregation in percent (MA%) are summarized in Table 1 . The mean CVs of the individual reagents ranged from 1.4 to 4.6% for the MA% value. The highest CV for TXRA observed in a series of 6 × 5 tests on five individual PRP samples with all reagents was 6.7% with ARA in one sample. The mean CV over all reagents and samples in this series was 2.8% on TXRA and 3.3% on PAP-8. TXRA generates very reproducible results with all reagents, but also the manual method performed by an experienced technician and with the instrument and reagents used in this study indicates high reproducibility. Precision values for slope and area under the curve (AUC) are given in Table 1 in the supplement. Mean CV over all reagents and samples was 6.9% for TRXA and 7.5% on PAP-8 for slope, and 3.2 versus 2.9% for AUC, respectively, indicating similar precision for these calculated results. Example readings from TXRA for all agonists are presented in the supplement ( Fig. 1 ).

### Reference Range

**Fig. 2:**
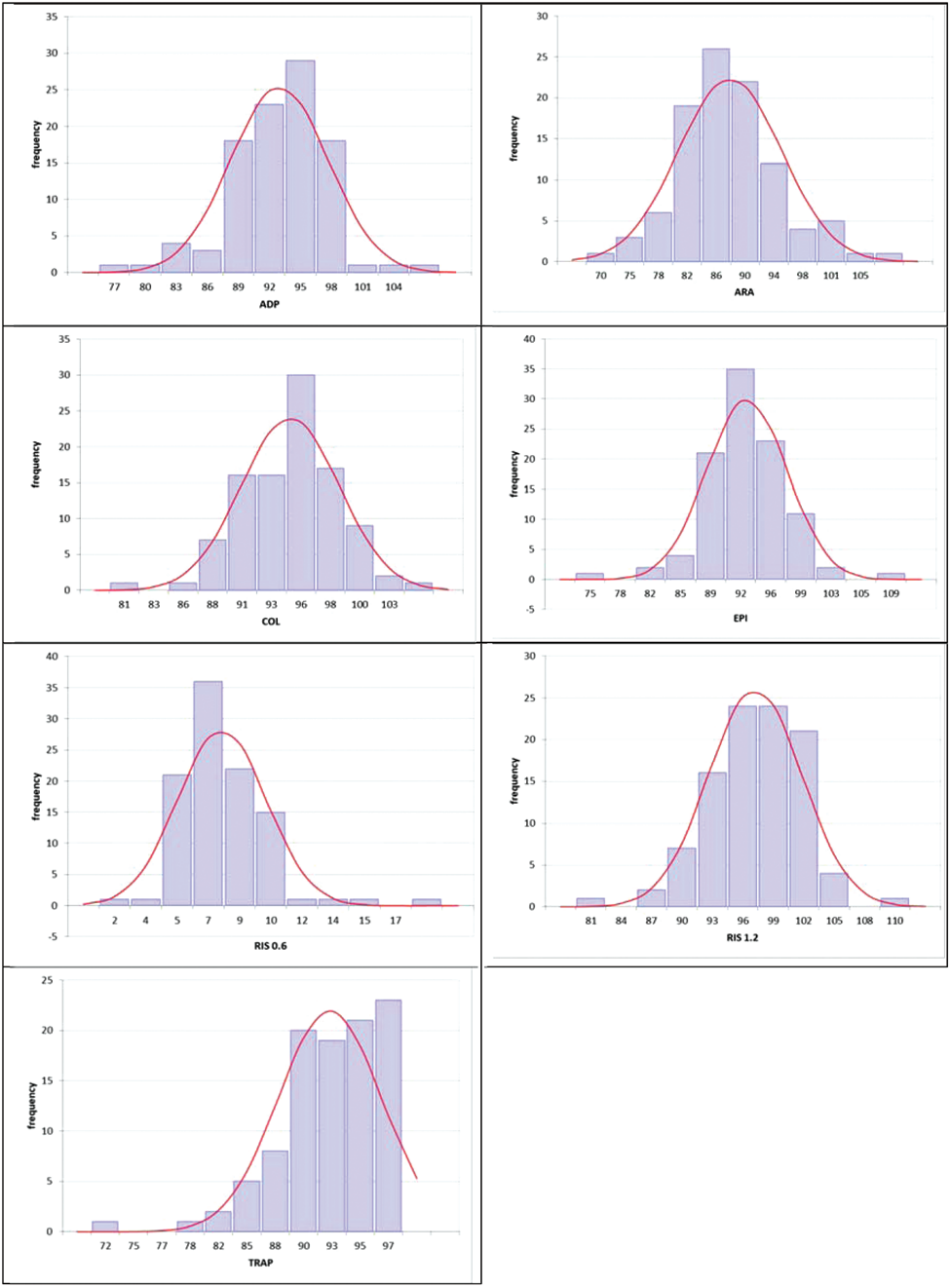
Normal range distribution histograms (TXRA, MA%). The distribution of MA% values obtained on TXRA is shown for MA% for all reagents used in this study.

The normal ranges for all reagents were tested in PRP samples from 50 female and 50 male healthy blood donors, aged between 18 and 65 years. All donors were free of medication, had no history of hemostatic problems, and their full blood counts were normal. Their results for MA% are summarized in Table 2 . Mean and median values were similar for all reagents on TXRA. The MA% results on TXRA obtained in male donors were slightly higher than in females, and also the distribution was somewhat wider ( Fig. 1A-C ), but differences did not reach statistical significance. Results obtained on TXRA showed upper (+2 standard deviation [SD]) and lower (−2SD) limits of the reference range that were slightly higher than on PAP-8, but method comparison shows a linear relationship with excellent correlation ( Fig. 1D ). The distribution for each platelet agonist is shown in Fig. 2 . Mean and median values were very similar in normal blood donors. TRAP 10 µM lead to almost complete aggregation on the TXRA in many normal samples. All other reagents showed a normal distribution. Low RISTO (0.6 mg/mL) gave values between 2 and 11.5 MA% (2SD range) on the TXRA, but 0 to 5% (2SD range) on the PAP-8 (data not shown). This probably reflects minor differences in reactivity by different mechanical features of the two instruments or other factors.

**Table 2:** Reference ranges for MA%. All healthy individuals ( *n* = 100)

	Thrombomate XRA						PAP-8		
	Reference: PPP			Reference: VPPP			Reference: PPP		
	All						All		
	Median	Mean	2SD range	Median	Mean	2SD range	Median	Mean	2SD range
ADP	92.2	91.6	82.2–101.0	87.8	87.2	82.7–91.6	82.0	81.5	69.1–93.9
ARA	85.7	86.1	72.3–99.8	82.4	82.8	77.5–88.0	82.5	82.8	70.5–95.0
COL	93.8	93.5	85.4–101.7	88.7	87.8	83.8–91.8	82.5	83.7	71.1–96.3
EPI	91.7	91.2	82.0–100.4	87.6	86.4	82.1–90.6	82.0	81.1	68.1–94.1
RISTO 0.6	6.4	6.7	2.0–11.5	4.4	4.7	2.8–6.8	1.0	1.7	0–4. 8
RISTO 1.2	95.8	95.4	86.3–104.5	90.1	89.5	85.8–93.1	80.0	80.5	70.5–90.4
TRAP	91.6	91.1	82.0–100.3	88.1	90.1	81.6–91.5	81.0	81.0	68.4–93.6

Abbreviations: ADP, adenosine diphosphate; ARA, arachidonic acid; COL, collagen; EPI, ephinephrine; MA%, maximum aggregation; PPP, platelet poor plasma; RISTO, ristocetin; SD, standard deviation; TRAP, thrombin receptor activating peptide (Ser-Phe-Leu-Leu-Arg-Asn); VPPP, virtual platelet poor plasma.

**Table 3:** Reference ranges for MA%. Gender-specific normal ranges (TXRA, against PPP)

TXRA	Females ( *n* = 50)			Males ( *n* = 50)		
	Median	Mean	2SD range	Median	Mean	2SD range
ADP	92.4	91.8	83.2–100.4	91.8	91.5	81.2–101.7
ARA	85.1	86.2	72.6–99.7	86.0	86.0	72.0–100.0
COL	93.6	93.5	86.1–100.9	93.9	93.5	84.6–102.5
EPI	91.7	91.7	83.9–99.5	91.4	90.8	80.4–101.2
RISTO 0.6	6.2	6.7	1.5–11.9	6.5	6.7	2.5–11.0
RISTO 1.2	95.4	95.7	87.9–103.4	96.0	95.2	84.9–105.6
TRAP	91.4	91.1	81.5–100.6	92.0	91.1	82.3–100.0
*PAP-8*						
ADP	80.0	80.6	69.4–91.8	82.5	82.5	69.2–95.8
ARA	81.0	81.4	72.4–90.3	83.5	84.1	69.7–98.6
COL	82.0	82.7	72.8–92.7	83.0	84.7	70.0–99.3
EPI	80.0	80.1	68.0–92.2	82.0	82.1	68.5–95.8
RISTO 0.6	1.0	1.5	0–4.6	1.5	1.8	0–4.9
RISTO 1.2	80.0	79.8	72.2–87.4	81.0	81.1	69.4–92.9
TRAP	79.	79.7	69.8–89.7	81.0	82.2	67.8–96.6

Abbreviations: ADP, adenosine diphosphate; ARA, arachidonic acid; COL, collagen; EPI, ephinephrine; MA%, maximum aggregation; PPP, platelet poor plasma; RISTO, ristocetin; TRAP, thrombin receptor activating peptide (Ser-Phe-Leu-Leu-Arg-Asn); TXRP, Thrombomate XRA; VPPP, virtual platelet poor plasma.

### Patient Data with Autologous Platelet-Poor Plasma

**Fig. 3:**
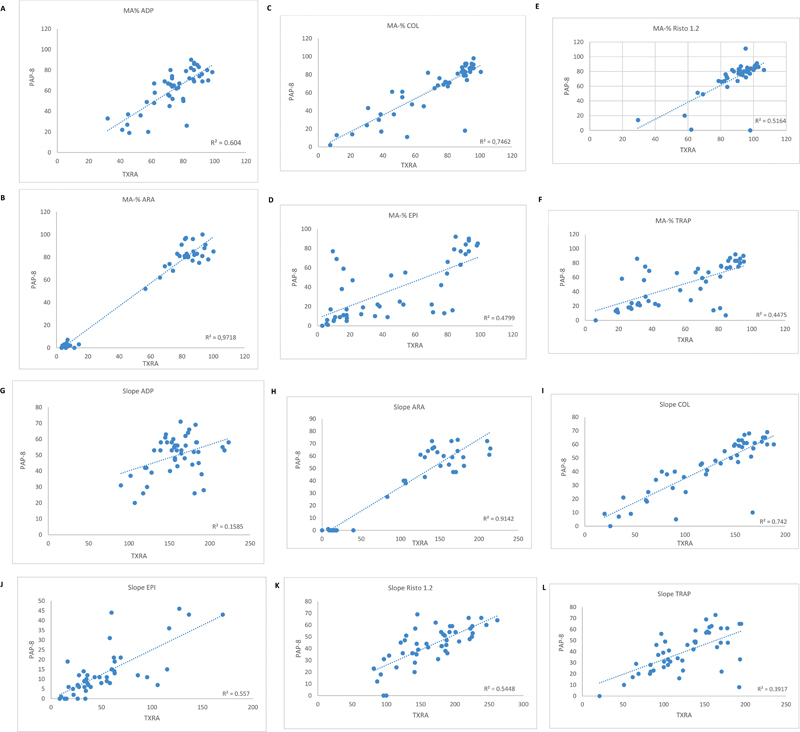
Method comparison of individual reagents between TXRA and PAP-8 in 47 patients: MA% and slope values. ( **A–F** ) maximum aggregation values in percentage, and ( **G–L** ) slope values (in instrument specific dimensions). *Y* -axis shows PAP-8, and X-axis shows TXRA results. *R* ^2^ , Pearson's correlation coefficient. Results obtained with RISTO 0.6 are not shown graphically because of the weak signal generated.

The individual patient data ( Fig. 3 ) indicated principal agreement between TXRA and PAP-8 for both slope and MA% values for all reagents. The closest correlation for MA% was achieved for ARA ( *r* ^2^ = 0.978), and the correlation for COL, ADP, and RIS was also satisfactory ( *r* ^2^ = 0.746, 0.604, and 0.516, respectively). EPI and TRAP showed some scatter in individual paired data. As expected, RIS 0.6 showed only marginal responsiveness in all except one patient. This patient with Von Willebrand disease type 2B showed significant reactivity with RIS 0.6 (PAP-8: 81% MA, TXRA: 95.8% MA) on both systems, with 87 and 100% MA with RIS 1.2. Of *n* = 20 patients taking aspirin, all 20 were identified on PAP-8 and TXRA. Their median MA% was 2 (range, 0–7) on PAP-8 and 5.95 (range, 3.3–14.3) on TXRA for ARA. In total, out of 47 patients considered to have a platelet function defect, 42 had at least one test below the 2SD range on PAP-8, and 42 had at least one test below the 2D range on TXRA. Of these, 40 patients were identified with both methods.

Passing–Bablok analysis demonstrates a linear relationship over the whole range of MA% data.

**Fig. 4:**
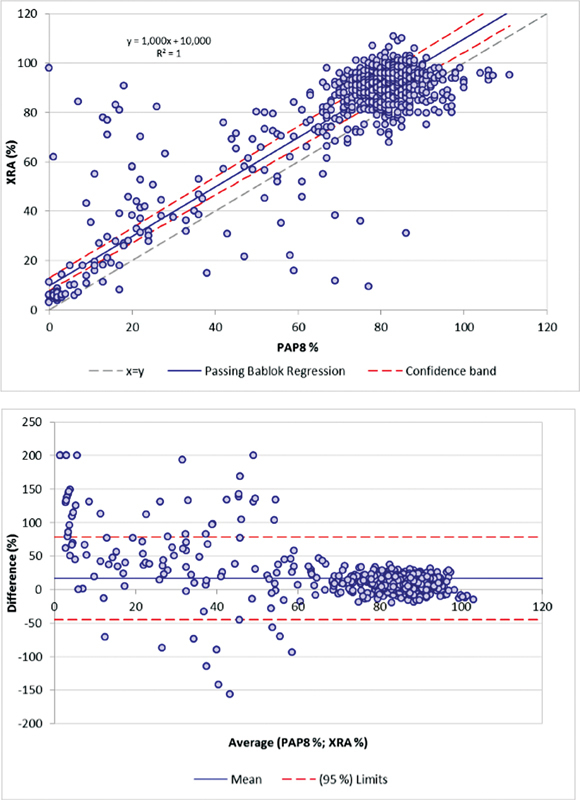
Method comparison using MA% for *n* = 47 patients and *n* = 100 healthy individuals tested on TXRA against PAP-8 with identical reagents. ( **A** ) Passing–Bablok analysis and ( **B** ) Bland–Altman plot. Data represent 1,029 individual results from healthy controls ( *n* = 100, seven reagents) and 47 patients ( *n* = 47, seven reagents).

Fig. 4 shows the combined method comparison of normal and patients. Bland–Altman analysis shows no identity, but very similar diagnostic information with both systems. Method comparison for area under the curve and slope is presented in the supplement, Fig. 2 .

### Results Obtained with Virtual Platelet-Poor Plasma

**Fig. 5:**
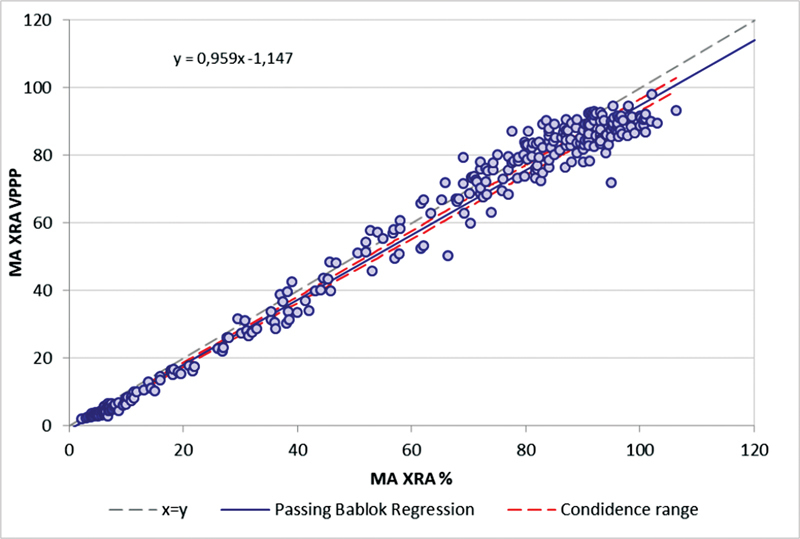
Comparison between TXRA PPP (= traditional LTA) against the VPPP method (without autologous PPP). Individual results from 47 patients tested with seven different reagents (329 individual data points) are shown with either PPP or VPPP as the reference. Pearson's correlation coefficient ( *R* ^2^ ) is 0.849.

TXRA employs artificial intelligence to reconstruct the optical properties of a fictive autologous PPP, called the VPPP. This approach allowed to recalculate all results that have been obtained against autologous PPP retrospectively against VPPP based on PRP data stored in the database. This virtual PPP corrects the spectral properties of a PRP an added reagent that it resembles autologous PPP by analysis of its spectral properties according to a novel complex proprietary algorithm. The analysis of healthy controls ( *n* = 100) and patients with confirmed or suspected platelet function defects ( *n* = 47, Fig. 5 ) demonstrates that there is little scatter between the set of data obtained with PPP and VPPP. Calculating the reference ranges with VPPP in comparison to PPP shows very similar ranges than obtained against PPP ( Fig. 5 B ), however with a few percent lower MA% values.

## Discussion

TXRA is an automated LTA system reporting results that correlate well with an established manual method. LTA is typically performed as a single determination, demanding high precision. In our study, TXRA showed excellent precision with all reagents, comparable to data obtained on the manual instrument, for which the coefficient of variability (CV) was only slightly higher. The consistent low CV on the manual instrument was probably related to the fact that one individual, very experienced technician operated it throughout the study, strictly following standard operation procedures with respect to PRP resting time, controlled PRP inverting before testing, and skilled pipetting. Precision for manual LTA obtained in this study may not be representative for laboratories in which several and/or less specialized technicians are involved, which likely leads to slightly divergent approaches to the test. In contrast, the automated device offers permanently standardized operation conditions. Another potential contributing factor for excellent CVs may also be the use of preset, fixed concentrations of agonists, which avoids variability due to predilution steps or freeze-and-thaw cycles. Good precision was also reported for LTA automated on coagulation analyzers. 19 Our precision data confirm that LTA represents a highly reproducible method if external influences are reduced to a minimum. A potential limitation of our data is that all samples for the precision study were from healthy blood donors and did not include abnormal platelet counts or clinically relevant, patient-related confounded variables such as, hyperlipidemia, hyperbilirubinemia, hemolysis, or low von Willebrand factor. 20

Our analysis of results focuses primarily on the most widely used parameter, maximum aggregation in percent (MA%). Other calculated parameters of the aggregation signatures were thoroughly inspected. In most cases, turbidity signatures were very similar, with some variability in individual cases, which we especially observed with EPI and TRAP (see supplement, Table 1 ). Unfortunately, comparing quantitative disintegration data was not expedient since observation time at TXRA was preset (6 minutes in this study) and clearly shorter than on the PAP-8 device (15 minutes). However, the observation can be prolonged also on TXRA for detecting late disintegration. Rapid disintegration in a patient sample is of course visible already in the 6 minutes observation period (see example in the supplement, Fig. 1 ).

The normal range for MA% showed a tendency to slightly higher values obtained with TXRA. MA% for all reagents showed normal distribution and minor variances between males and females, not reaching statistical differences. Low RISTO (0.6 mg/mL) displayed little differences between the TXRA and PAP-8. While PAP-8 did not generate measurable aggregation in several normal samples, TXRA showed explicit values in the range between 2 and 11.5% MA. This may be related to the mechanical factors: while PAP-8 works with round cuvettes and stir bars, TXRA has a flat cuvette, and mixing is achieved by lateral movement of a steel ball by magnetic forces on a rail-like structure on the bottom of the cuvette. The difference, however, could also be caused by the individual calculation algorithms for MA% used in the two devices. It remains open if an apparently enhanced resolution in this range by TXRA supports the analysis of patients with von Willebrand's disease, specifically in type 2B or platelet type von Willebrand, or in Bernard Soulier syndrome. Results in one patient with VWS type 2B with both systems were comparable. Aggregometry results from unselected patients in general showed good correlation. The degree of correlation varied depending on the agonists used. The agreement between TXRA and the manual device with several agonists was very close for slope, AUC, and MA%, while there was some scatter with EPI and TRAP. For these two agonists, results showed a very similar trend but are not identical. This may reflect minor differences between the mechanical factors, the optical systems, and software in the two systems. Differences in EPI and TRAP between TRXA and the manual device were not observed in healthy subjects and may reflect a different sensitivity to minor changes in platelet reactivity. A time-dependent change of reactivity in the samples, when not tested at exactly the same time on both devices, may contribute. A previous study between two instruments of the same brand as the manual system used in this study showed differences as well. 21 Since there is no established reference method for aggregometry, results cannot be checked for “true” accuracy.

A novel aspect of this study is performing LTA without a reference by measuring PRP against PPP. Interestingly, we could demonstrate here that TXRA enables LTA without the use of autologous PPP by implementing an artificial intelligence-based approach on TXRA. Results correlate strongly with the classical method with autologous PPP. Beyond a relevant reduction in workload, this novel feature has also the potential of reducing total blood volume, an advantage for children, but also for patients in general.

In conclusion, this study shows that full automation of LTA with TXRA is feasible, precise, and even possible in the absence of autologous PPP.

## Notes

**What is Known on this Topic?**

- Light transmission aggregometry is considered the gold standard for platelet function testing.
- It is a labor-intensive test involving multiple manual steps.
- Additional platelet-poor plasma is required to calibrate the instrument.

**What does this Paper Add?**

- A fully automated light transmission aggregometry device delivers precise measurements.
- Automated aggregometry results correlate well with the manual method and make the test almost user independent.
- Instead of platelet-rich plasma, a virtual reference via artificial intelligence can be used to calibrate the instrument and run the tests.
